# Cigarette smoke increases TLR4 and TLR9 expression and induces cytokine production from CD8^+ ^T cells in chronic obstructive pulmonary disease

**DOI:** 10.1186/1465-9921-12-149

**Published:** 2011-11-09

**Authors:** Jessica Nadigel, David Préfontaine, Carolyn J Baglole, François Maltais, Jean Bourbeau, David H Eidelman, Qutayba Hamid

**Affiliations:** 1 Meakins-Christie Laboratories, Faculty of Medicine, McGill University, Montreal, Qc, Canada; 2 Respiratory Division, Research Institute of McGill University Health Centre, Montreal, Qc, Canada; 3 Respiratory Division, Laval University, Quebec, Qc, Canada

**Keywords:** COPD, Toll-like receptors, CD8^+ ^T cell, cigarette smoke, cytokine

## Abstract

**Background:**

Cigarette smoke is a major risk factor for chronic obstructive pulmonary disease (COPD), an inflammatory lung disorder. COPD is characterized by an increase in CD8^+ ^T cells within the central and peripheral airways. We hypothesized that the CD8^+ ^T cells in COPD patients have increased Toll-like receptor (TLR) expression compared to control subjects due to the exposure of cigarette smoke in the airways.

**Methods:**

Endobronchial biopsies and peripheral blood were obtained from COPD patients and control subjects. TLR4 and TLR9 expression was assessed by immunostaining of lung tissue and flow cytometry of the peripheral blood. CD8^+ ^T cells isolated from peripheral blood were treated with or without cigarette smoke condensate (CSC) as well as TLR4 and TLR9 inhibitors. PCR and western blotting were used to determine TLR4 and TLR9 expression, while cytokine secretion from these cells was detected using electrochemiluminescence technology.

**Results:**

No difference was observed in the overall expression of TLR4 and TLR9 in the lung tissue and peripheral blood of COPD patients compared to control subjects. However, COPD patients had increased TLR4 and TLR9 expression on lung CD8^+ ^T cells. Exposure of CD8^+ ^T cells to CSC resulted in an increase of TLR4 and TLR9 protein expression. CSC exposure also caused the activation of CD8^+ ^T cells, resulting in the production of IL-1β, IL-6, IL-10, IL-12p70, TNFα and IFNγ. Furthermore, inhibition of TLR4 or TLR9 significantly attenuated the production of TNFα and IL-10.

**Conclusions:**

Our results demonstrate increased expression of TLR4 and TLR9 on lung CD8^+ ^T cells in COPD. CD8^+ ^T cells exposed to CSC increased TLR4 and TLR9 levels and increased cytokine production. These results provide a new perspective on the role of CD8^+ ^T cells in COPD.

## Introduction

Chronic obstructive pulmonary disease (COPD) is a leading cause of morbidity and mortality worldwide [1], with more than 80% of COPD cases caused by cigarette smoking [2]. Chronic inflammation observed in COPD is characterized by pro-inflammatory cytokine production and recruitment of several cell types to the lungs, including cells of the innate immune response, such as neutrophils and macrophages [3], as well as those of adaptive immune response, namely T and B lymphocytes [4,5]. CD8^+ ^T cells are regarded as a hallmark cell of COPD, and are increased in both the central [6] and peripheral [7] airways of COPD patients. CD8^+ ^T cells found within the airways are generally located within the submucosa and invading the epithelium [8,9]. Unfortunately, the role of CD8^+ ^T cells in COPD and the mechanisms by which they are recruited to the lung are still generally unknown. While it can be speculated that these cytotoxic T cells promote injury to the already damaged lung, they could also contribute towards protecting the lung by sensing invading microbes and using their cytotoxic abilities to eliminate infected cells.

Toll-like receptors (TLR), a key component of the innate immune system, sense foreign microbes via pathogen-associated molecular patterns. Although largely found on innate immune and structural cells [10,11], TLRs are also present on T cells, thereby contributing to the adaptive immune response [12-15]. TLR4, which recognizes gram-negative bacteria, and TLR9, which binds unmethylated CpG motifs, are two well-studied TLRs. Activation of TL4 or TLR9, results in signal transduction cascades involving downstream pathways including nuclear factor of kappa B (NF-κB) and JUN N-terminal kinase (JNK) [16]. This ultimately results in the production of inflammatory cytokines such as IL-1β, IL-6, IL-8, TNFα and IL-10 which can modulate inflammatory responses [17-19].

There is growing interest in the potential role of TLRs in COPD pathogenesis, [20,21] including the relationship between cigarette smoke exposure and the expression of TLRs on epithelial cells [22,23]. In our study, we investigated the expression of TLR4 and TLR9 on CD8^+ ^T cells, an important cell type in COPD pathogenesis. We report for the first time increased expression of TLR4 and TLR9 on CD8^+ ^T cells in lung tissue of COPD patients compared to control subjects. Moreover, our data further demonstrates that cigarette smoke exposure induces TLR4 and TLR9 expression on CD8^+ ^T cells, which results in increased production of cytokines, including IL-1β, IL-6, IL-10, IL-12p70, TNFα and IFNγ. Cigarette smoke activation of TLRs on CD8^+ ^T cells and the resulting increased cytokine production represents a mechanism by which CD8^+ ^T cells can contribute to the pathogenesis of COPD.

## Materials and methods

### Study subjects

Endobronchial biopsies from eight COPD patients and five aged-matched control subjects were received from the Tissue Bank of the Respiratory Health Network of the FRSQ, MUHC site. Peripheral blood was obtained from nine COPD patients and eight control subjects recruited at the Montreal Chest Institute. Each participant gave a total of 20 ml of peripheral blood and underwent spirometry. Control subjects represented healthy volunteers, either non-smokers or ex-smokers, with normal lung function. Participant details can be found in Table 1. This study was reviewed and approved by The Biomedical C Research Ethics Board of the Montreal Chest Institute, and written informed consent was obtained from all subjects.

**Table 1:** Characterization of smoking status and demographics of COPD patients and control subjects

		Control	COPD
***Biopsy***		(n = 5)	(n = 8)
	Age (years)	54.8 ± 7.16	65.0 ± 17.33
	Sex (M:F)	4:1	6:2
	FEV_1 _(L)	3.69 ± 0.89	2.31 ± 0.84
	FEV_1_/FVC (ratio)	0.83 ± 0.01	0.56 ± 0.08
	GOLD Stage (I/II/III/IV)	0/0/0/0	3/3/2/0
	Smoking History		
	Pack Years	3.4 ± 4.77	38.25 ± 14.76
	Current smokers	0	5
	Ex-smokers	2	3
	Non-smokers	3	0
			
***Blood***		(n = 8)	(n = 9)
	Age (years)	59.86 ± 7.15	64.63 ± 4.44
	Sex (M:F)	5:3	6:3
	FEV1 (L)	3.14 ± 0.63	1.21 ± 0.570
	FEV_1_/FVC	0.79 ± 0.03	0.59 ± 0.25
	GOLD Stage (I/II/III/IV)	0/0/0/0	0/4/4/0
	Smoking History		
	Pack Years	13.59 ± 24.33	62.06 ± 29.67
	Current smokers	0	1
	Ex-smokers	4	8
	Non-smokers	4	0

FEV_1_: Forced expiratory volume in 1 second, FVC: Forced vital capacity

### Immunostaining

Endobronchial biopsies were taken from the segmental and subsegmental carinas of the right upper lobe and immediately fixed in paraformaldehyde. Biopsies were then embedded in paraffin and cut into 5 μm sections. The sections were de-paraffinized in xylene, rehydrated in ethanol and washed with PBS. Antigen retrieval was performed using citrate buffer (pH 6); the sections were permeabilized with 0.2% Triton X-100 (in PBS) and then incubated with 5% hydrogen peroxide (in PBS). After washing, the sections were blocked with Universal Blocking Solution (Dako, Burlington, ON) for 30 minutes. Slides were incubated overnight at 4°C with a rabbit polyclonal IgG antibody against TLR4 or TLR9 (1.25 μg/ml; Abcam, Cambridge, MA). Slides were then incubated with a biotinylated secondary antibody raised in goat (1:100), followed by incubation with SA-HRP (Vector Laboratories, Burlington, ON). The immunostainings were developed using the Liquid DAB+ Substrate Chromogen System (Dako) according to the manufacturer's instructions and counterstained with hematoxylin and lithium carbonate. Within the submucosa, the number of immune cells positive for TLR4 or TLR9 were quantified and expressed as the number of positive immune cells per square millimeter of subepithelium.

To co-localize TLR4 or TLR9 to CD8^+ ^cells, double immunofluorescence was performed on endobronchial biopsies embedded in OCT. Blocks were cut into 5 μm sections and fixed with 70% ethanol for 30 seconds. After washing with PBS, sections were permeabilized and blocked as above. Sections were incubated overnight at 4°C with a monoclonal IgG1κ CD8 antibody (clone C8/144B, Dako) at a concentration of 2 mg/ml. After washing, the sections were incubated with a rabbit anti-mouse AlexaFluor 555 (Invitrogen, Carlsbad, CA) secondary antibody for 1 hour in the dark at a concentration of 5 μg/ml. Next, the sections were incubated with either the TLR4 or TLR9 antibody as described above for 2 hours at room temperature. A goat anti-rabbit AlexaFluor 488 (Invitrogen) secondary antibody was used at a concentration of 5 μg/ml for 1 hour in the dark. Sections were then washed and mounted using PermaFluor™ Aqueous Mounting Medium (Thermo Scientific, Waltham, MA). The absolute numbers of the single- and double-stained cells were counted and results were expressed as the percentage of CD8^+ ^T cells expressing TLR4 or TLR9. All slides were imaged using the Image-Pro Plus 6.2 system (Media Cybernetics, Bethesda, MD).

### Peripheral blood mononuclear cell isolation and culture

Peripheral blood mononuclear cells (PBMC) were separated from the peripheral blood using Ficoll-Paque Plus (GE Healthcare, Baie D'Urfé, QC). Cells were maintained in RPMI-1640 medium (Thermo Scientific) supplemented with 10% v/v fetal bovine serum, L-glutamine, penicillin, streptomycin, sodium pyruvate, and HEPES and incubated at 37°C with 5% CO_2_.

### Flow cytometry cell staining of PBMC

PBMCs were first incubated with intravenous immunoglobulin to prevent any non-specific binding. Cells were then stained with an AlexaFluor 488-conjugated mouse IgG2a antibody against TLR4, clone HTA125 (AbDSerotec, Kidlington, Oxford), or with a FITC-conjugated mouse IgG2a antibody against TLR9, clone 5G5 (Abcam). All cells were stained with a CD8 antibody conjugated to APC, mouse IgG1κ, clone RPA-T8 (BD Pharmingen™, Mississauga, ON,) and a CD3 antibody conjugated to PE, mouse IgG1κ, clone UCHT1 (BD Pharmingen™). All corresponding isotype control antibodies were used. Cells stained for TLR9 were permeabilized with Cytofix/Cytoperm (BD Pharmingen™). A total of 25,000 events were acquired by flow cytometry (FACSCalibur, BD Bioscience) and analyzed using CellQuest™ Pro Software. Data are presented as the percentage of positively stained cells for each subject.

### CD8^+ ^T cell isolation and cigarette smoke condensate treatment

CD8^+ ^T cells were isolated from PBMCs using the CD8^+ ^T Cell Isolation Kit (Miltenyi Biotec, Bergisch Gladbach, DE) according to the manufacturer's protocol. Briefly, PBMCs were suspended in buffer (PBS containing 0.5% w/v BSA and 2 mM EDTA, pH 7.2) and incubated with a biotin-antibody cocktail containing antibodies against CD4, CD15, CD16, CD19, CD34, CD36, CD56, CD123, TCR γ/δ and CD235a for 10 minutes at 4°C. Next, a microbead-conjugated antibody against CD14 and biotin was added for 15 minutes at 4°C. The cell suspension was separated using LS columns with the QuadroMACS™ (Miltenyi Biotec) cell separator. The enriched CD8^+ ^T cells passed through the column and were collected. The CD8^+ ^T cell purity was ≥ 95% as confirmed by flow cytometry staining (data not shown).

Once purified, the CD8^+ ^T cells were incubated with cigarette smoke condensate (CSC), which was received as a gift from Imperial Tobacco Canada, generated as previously reported [24]. The CD8^+ ^T cells were treated with CSC at either 10 or 50 μg/ml for 24 hours. CD8^+ ^T cells were also pretreated with or without a TLR4 neutralizing antibody (10 μg/ml; clone HTA125, eBioscience, San Diego, CA) or chloroquine (20 μg/ml; Invivogen, San Diego, CA) for 1 hour followed by CSC at 50 μg/ml for 24 hours. Cells receiving DMSO were used as the control as DMSO was the solvent for the CSC.

### Protein quantification and immunoblotting

CD8^+ ^T cells were lysed in 100 μL of lysis buffer (50 mM Tris-HCl pH 7.5, 1 mM EGTA, 1 mM EDTA, 1% (v/v) Triton x-100, 1 mM sodium orthovanadate, 5 mM sodium pyrophosphate, 50 mM sodium fluoride, 0.27 M sucrose, 5 mM sodium pyrophosphate decahydrate and protease inhibitors). Protein concentrations were quantified using the BCA Protein Assay Kit (Thermo Scientific) according to the manufacturer's instructions. Fifty micrograms of protein were boiled and separated on a 10% Pro-Pure Next Gel with Pro-Pure Running Buffer (Amresco, Solon, OH). After transferring proteins to nitrocellulose, membranes were blocked for 1 hour at room temperature in Odyssey Blocking Buffer (Li-Cor Biosciences, Lincoln, NE). Blots were then incubated with anti-TLR4 antibody (H-80; Santa Cruz, Santa Cruz, CA) at a concentration of 0.2 μg/ml or anti-TLR9 antibody (Abcam) at a concentration of 1 μg/ml overnight at 4°C. Goat anti-rabbit IgG (DyLight™800, Thermo Scientific) antibody was applied for 1 hour in the dark at room temperature (1:15,000). The signal was detected and quantified using a Li-Cor Odyssey imaging system. All samples were normalized to GAPDH (Millipore, Billerica, MA) and expressed as a ratio relative to the DMSO sample.

### Cytokine protein quantification

Supernatants were collected 24 hours after CD8^+ ^T cells were treated with CSC and stored at -80°C until used. The Human Pro-Inflammatory 7-Plex Tissue Culture Kit was purchased from Meso Scale Discovery (Gaithersburg, MD) and used according to the manufacturer's protocol. The kit allowed for the quantification of IL-1β, IL-6, IL-8, IL-10, IL-12p70, TNFα, and IFNγ from the cell supernatants using the SECTOR^® ^Imager 2400. The lower limits of detection for this plate were as follows: IL-1β, 0.0199 pg/ml; IL-6, 0.0158 pg/ml; IL-8, 0.0125 pg/ml; IL-10, 0.0794 pg/ml; IL-12p70, 0.0199 pg/ml; TNFα, 0.3162 pg/ml, IFNγ, 0.0 pg/ml. The upper limit of detection was 10,000 pg/ml for all cytokines.

### Statistical analysis

For statistical analysis between two groups, a t-test was used. Comparison between more than two groups was performed by ANOVA, followed by a Bonferonni multiple comparison test. A p-value of < 0.05 was considered statistically significant (*p < 0.05; **p < 0.01; ***p < 0.0001). Data are expressed as mean ± standard error of mean.

## Results

### TLR4 and TLR9 are expressed in COPD patients and control subjects

**Figure 1:**
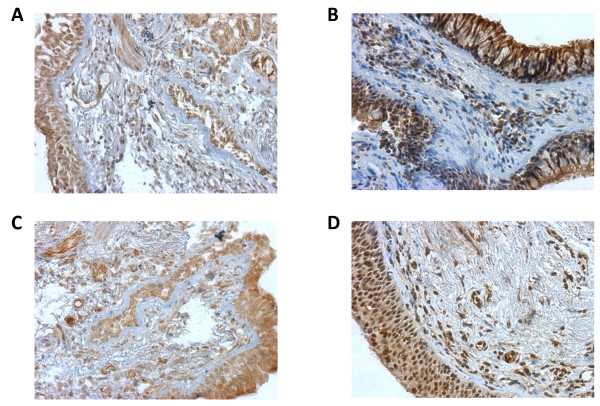
**TLR4 and TLR9 are expressed in lung tissue**. Endobronchial biopsy tissue sections from COPD patients and control subjects were processed by immunohistochemistry as described in Materials and Methods. Representative examples show similar immunoreactivity for TLR4 in the epithelium and inflammatory cells of control subjects (A) and COPD patients (B). TLR9 was also expressed at comparable levels in the epithelium and inflammatory cells in healthy controls (C) and COPD patients (D). All magnifications are 200×.

**Figure 2:**
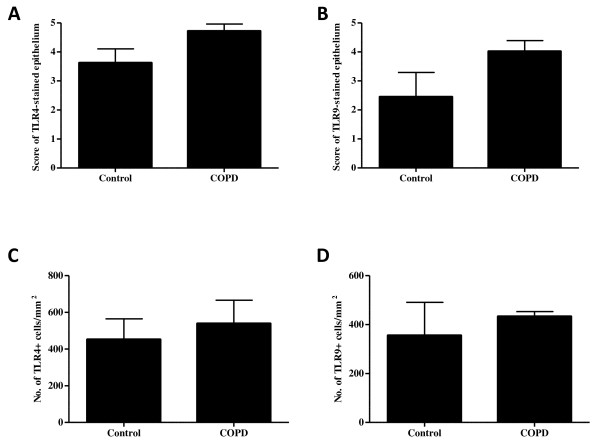
**Quantification of TLR4 and TLR9 in COPD and control subjects**. The degree of epithelial staining of TLR4 (A) and TLR9 (B) were scored according to a scale (1 = no staining; 5 = 100% staining) following immunostaining. The numbers of the positively stained immune cells were also counted, and the area of the tissue was measured. There was no significant difference in the percentage of TLR4- (A) or TLR9- (B) stained epithelium between the two groups. There was also no significant difference in the total number of inflammatory cells expressing TLR4 (C) and TLR9 (D) between the control group and COPD patients, when expressed as cells/mm^2 ^tissue.

Activation of TLRs can lead to the production of pro-inflammatory mediators, many of which are increased in COPD. However, there is little information on the expression of TLR4 or TLR9 in the lungs of COPD patients. Therefore, we first examined the expression of TLR4 and TLR9 by immunohistochemistry in endobronchial biopsies from COPD patients and control subjects. TLR4 is expressed in the airway epithelium and inflammatory cells in both healthy controls (Figure 1A) as well as COPD patients (Figure 1B). In addition, immunoreactivity of TLR9 was also found in the epithelium and the immune cells within the subepithelial layer in both control subjects (Figure 1C) and patients with COPD (Figure 1D). The epithelium was scored according to the extent of staining, thus the percentage of epithelial cells showing positive immune reactivity. There was no significant difference between controls and COPD patients in the percentage of the epithelial cells expressing TLR4 (Figure 2A) or TLR9 (Figure 2B). In order to further quantify the staining in the subepithelium, the number of positive immune cells per square millimeter were counted from both COPD and control biopsies for each of the TLRs of interest. There was also no significant difference in the number of immune cells expressing TLR4 (Figure 2C) or TLR9 (Figure 2D) in COPD biopsies compared to control biopsies.

### Co-localization of TLR4 and TLR9 on lung CD8^+ ^T cells in COPD

**Figure 3:**
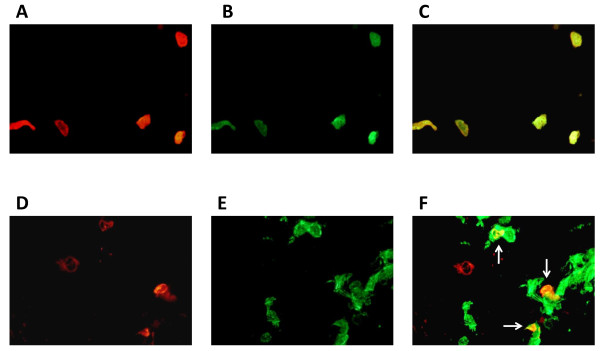
**Lung CD8^+ ^T cells express TLR4 and TLR9 in COPD patients**. Representative immmunofluorescent images from frozen endobronchial biopsies of COPD patients demonstrate the co-localization of CD8 (red fluorescence, panels A and D) and TL4 (green fluorescence, panel B) or TLR9 (green fluorescence, panel E). The merged images show CD8^+ ^T cells expressing TLR4 or TLR9 (arrows, panels C and F, respectively). All magnifications are 400×.

Although the total lung expression of TLR4 and TLR9 did not differ significantly between COPD patients and control subjects, we wanted to examine the expression of TLR4 and TLR9 more closely in lung CD8^+ ^T cells. Therefore, we performed immunofluorescence staining on endobronchial biopsies obtained from both COPD and control subjects. Slides were stained for the presence of CD8^+ ^T cells, (Figures 3A, D), and stained with either TLR4 (Figure 3B) or TLR9 (Figure 3E) antibodies. An overlay of the images shows co-localization of CD8 immunoreactivity and TL4 and TL9 positive cells (Figures 3C, F).

**Figure 4:**
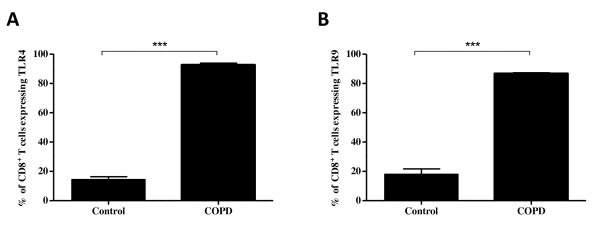
**Quantitative analysis of the number of CD8^+ ^T cells expressing TLR4 or TLR9 in endobronchial biopsies**. The absolute numbers of the singled-stained CD8^+ ^T cells were counted and compared to the absolute numbers of CD8^+ ^T cells expressing TLR4 or TLR9 from COPD patients (n = 6) and healthy controls (n = 5). The percentage of CD8^+ ^T cells expressing TLR4 (A) was significantly higher in COPD patients (92.76 ± 1.18) compared to control subjects (14.38 ± 2.00). The percentage of CD8^+ ^T cells expressing TLR9 (B) was also significantly increased in COPD patients (86.88 ± 0.51) compared to healthy controls (17.97 ± 3.74). Data are represented as mean ± SEM. *** indicate p-values < 0.0001.

Quantification of the staining was performed by counting the absolute numbers of single-stained CD8^+ ^T cells compared to the double-stained CD8^+ ^T cells, resulting in a percentage of CD8^+ ^T cells which express TLR4 and TLR9. In the lung tissue of COPD patients, there was a significant increase in the percentage of CD8^+ ^T cells expressing TLR4 (Figure 4A) (p < 0.0001) and TLR9 (Figure 4B) (p < 0.0001) when compared to control subjects. COPD patients had approximately 90% of their CD8^+ ^T cells co-expressing TLR4 and TLR9. The number of cells co-expressing CD8 and TLR4 or TLR9 was substantially lower in control subjects compared to COPD patients, with less than 20% of CD8^+ ^T cells expressing TLR4 or TLR9.

### Expression of TLR4 and TLR9 on peripheral blood mononuclear cells

**Figure 5:**
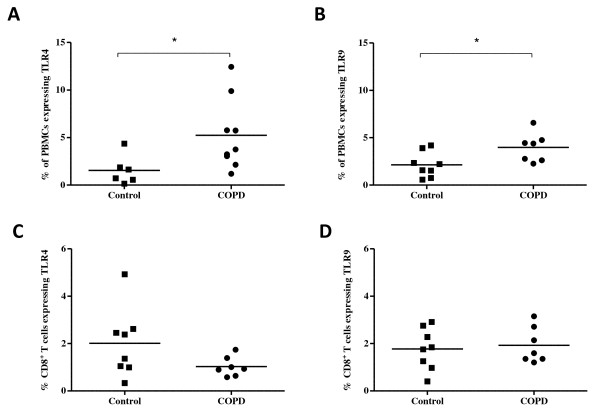
**The expression of TLR4 and TLR9 on CD8^+ ^T cells from peripheral blood**. Peripheral blood mononuclear cells (PBMC) were isolated from the peripheral blood of COPD patients (n = 7-9) and control subjects (n = 6-8). The percentage of cells positive for CD8, TLR4 and TLR9 were determined by flow cytometry. TLR4 (A) and TLR9 (B) expression in PBMCs was significantly increased in COPD patients compared to healthy controls. PBMCs were double-stained with CD8 and either TLR4 and TLR9 and expressed as the number of CD8^+ ^T cells expressing the receptor. There was no significant difference in the percent of CD8^+ ^T cells expressing TLR4 (C) or TLR9 (D) in COPD patients vs. healthy controls. Each data point represents an individual patient. * indicate p-values < 0.05.

Next, we wanted to investigate TLR4 and TLR9 expression in peripheral blood to observe if the expression was similar to that of the lung. Peripheral blood mononuclear cells (PBMC) were isolated and stained with TLR4 and TLR9 antibodies. Flow cytometry was used to determine that COPD patients had a significantly higher percentage of total PBMCs which expressed TLR4 (Figure 5A) (p < 0.05) and TLR9 (Figure 5B) (p < 0.05) compared to aged-matched control subjects. To then determine if the PBMCs expressing TLR4 or TLR9 included CD8^+ ^T cells, we stained the PBMCs with CD8, TLR4 or TLR9 and CD3. A CD3 antibody was used to ensure that the population of analyzed cells consisted of true T cells expressing CD8. It was found that the percentage of CD8^+ ^T cells which expressed TLR4 (Figure 5C) and TLR9 (Figure 5D) were not statistically different between COPD patients and control subjects. In addition, the overall expression of TLR4 and TLR9 was quite minimal on peripheral blood CD8^+ ^T cells. This indicates that, in contrast to what was observed in the lungs of COPD patients, CD8^+ ^T cells do not greatly contribute to TLR expression in the blood. While approximately 90% of the COPD lung CD8^+ ^T cells expressed these receptors, this was only observed in about 2% of peripheral blood CD8^+ ^T cells. This raises the possibility that there may be a factor in the lungs of COPD patients not found within in the blood, which can induce TLR4 and TLR9 expression on CD8^+ ^T cells. Furthermore, this factor would also have to be absent from lungs and blood of control subjects as these subjects had low TLR expression in general.

### Cigarette smoke condensate upregulates TLR4 and TLR9 protein

Our findings led us to speculate that cigarette smoke may be the contributing factor that can induce TLR4 and TLR9 expression on CD8^+ ^T cells. Cigarette smoke is the primary risk factor for COPD, and considering the location of CD8^+ ^T cells within the lung tissue, one would expect that these cytotoxic cells are exposed to smoke. Cigarette smoke exposure would also explain the lack of co-localization found in the peripheral blood. To test this hypothesis, CD8^+ ^T cells isolated from the peripheral blood of COPD patients were treated with cigarette smoke condensate (CSC) for 24 hours, mimicking the effects of cigarette smoke exposure to the lungs. Cytotoxicity was determined using trypan blue staining to demonstrate that greater than 80% of cells remained viable at concentrations of CSC up to 50 μg/ml (data not shown); this concentration approximates human exposures [25-27].

**Figure 6:**
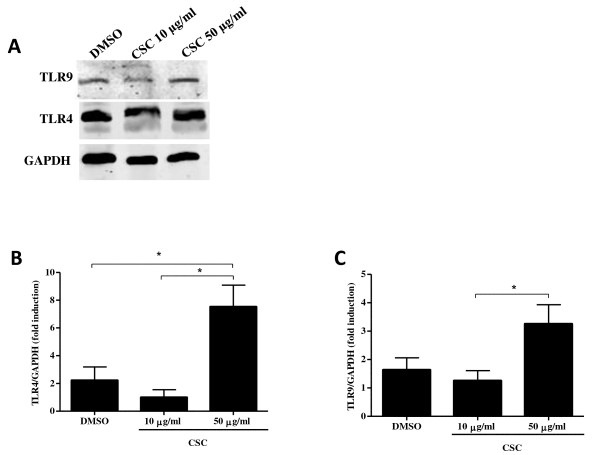
**TLR4 and TLR9 protein expression on CD8^+ ^T cells is upregulated by CSC**. CD8^+ ^T cells isolated from the peripheral blood of COPD patients (n = 4-5) and treated with CSC (10 μg/ml or 50 μg/ml) or DMSO for 24 hours. A representative western blot nitrocellulose membrane expressing TLR4, TLR9 and GAPDH (A). Western blot analysis revealed an increase in TLR4 (B) and TLR9 (C) protein expression with a CSC treatment of 50 μg/ml. Individual samples were normalized to GAPDH and then expressed as a relative ratio of treated compared to DMSO sample. Data are represented as mean ± SEM. * indicates p-values < 0.05.

TLR4 and TLR9 gene expression was assessed by qRT-PCR, and there was no significant change in TLR4 or TLR9 expression after 24 hours of CSC exposure (data not shown). A similar finding was previously observed when epithelial cells were treated with cigarette smoke [22]. In contrast, the expression of both TLR4 (Figure 6A, B) and TLR9 (Figure 6A, C) protein was significantly increased (p < 0.05) from baseline after a CSC exposure of 50 μg/ml, suggesting these receptors are post-transcriptionally regulated during CSC treatment.

### CSC induces cytokine production from CD8^+ ^T cells

**Figure 7:**
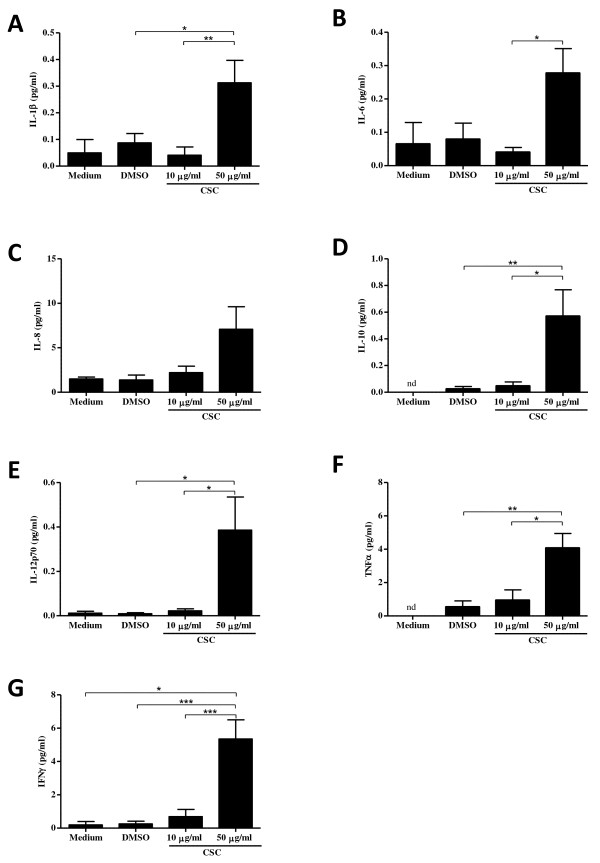
**CSC induces cytokine production from CD8^+ ^T cells**. CD8^+ ^T cells from COPD patients (n = 10) were treated with CSC (10 μg/ml or 50 μg/ml), DMSO, or medium for 24 hours. The supernatants were collected and cytokine expression was examined using the Human ProInflammatory 7-Plex Tissue Culture Plate from Meso Scale Discovery. There was no significant change in cytokine production between the untreated cells or those which were treated with DMSO. At 50 μg/ml of CSC treatment, there was a significant increase in cytokine expression for IL-1β (A), IL-10 (D), IL-12p70 (E), TNFα (F), and IFNγ (G) compared to DMSO. There was no change in IL-8 (C) expression. There was also a significant increase in cytokine production at 50 μg/ml compared to cells treated with 10 μg/ml of CSC for IL-1β (A), IL-6 (B) IL-10 (D), IL-12p70 (E), TNFα (F), and IFNγ (G). Data are represented as mean ± SEM. * indicates p-values < 0.05; ** indicates p-values < 0.01 and *** indicates p-values < 0.0001. nd = not determined.

Next, we wanted to investigate the activation of CD8^+ ^T cells by CSC to induce cytokine production. COPD- and CD8-associated cytokines were measured in the supernatants of peripheral blood CD8^+ ^T cells treated with CSC, DMSO or medium for 24 hours. There was no change in the cytokine production between the cells given media alone or those treated with DMSO. However, there was a significant increase in IL-1β, IL-10, IL-12p70, TNFα and IFNγ (Figures 7A, D-F) (p < 0.05) at the highest concentration (50 μg/ml) of CSC when compared to the DMSO-treated cells, while IL-6 was only significantly different between the two smoke concentrations (Figure 7B). There was no change in IL-8 production (Figure 7C). It should be noted that the concentrations of all cytokines released was relatively low, suggesting that CD8^+ ^T cells may lack the ability to secrete significant levels of cytokines in COPD. However, these cytokine values correspond to what is observed in the bronchoalveolar lavage fluid of patients with bronchiectasis, another type of chronic lung disease [28]. The significant increase in cytokine production at 50 μg/ml cannot be ignored and may be critical to the inflammatory setting. The increased expression of TLR4 and TLR9 on CD8^+ ^T cells, concomitant with CSC-induced release of pro-inflammatory cytokines, suggests that the CSC is activating the TLR pathways to induce the aforementioned cytokines.

### Blocking the TLR4 and TLR9 pathways inhibits cytokine production

**Figure 8:**
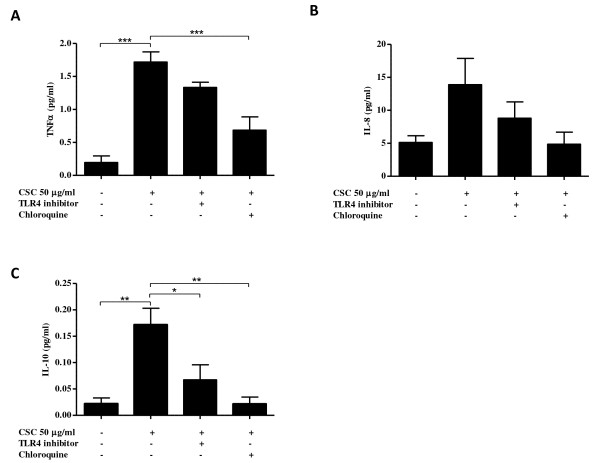
**Inhibitors of the TLR4 and TLR9 pathways reduce cytokine secretion from CD8^+ ^T cells**. CD8^+ ^T cells were incubated for 1 hour with or without a TLR4 neutralizing antibody (10 μg/ml) or chloroquine (20 μg/ml). Cells were then treated with either DMSO or CSC (50 μg/ml) for 24 hours. The supernatants were analyzed for cytokine expression using Human ProInflammatory 7-Plex Tissue Culture Plate. (A) Chloroquine significantly reduced CSC-induced TNFα expression. (B) IL-8 expression was not significantly increased by CSC, however chloroquine was able to reduce IL-8 levels back to baseline values. (C) Blocking the TLR4 and TLR9 pathways significantly attenuated the CSC-induced production of IL-10. Data are represented as mean ± SEM. * indicates p-values < 0.05; ** indicates p-values < 0.01 and *** indicates p-values < 0.0001.

To determine if the CSC is inducing cytokine production through the activation of TLR4 and TLR9, we utilized well-described inhibitors of TLR4 [29] and TLR9 [30]. One hour prior to CSC treatment (50 μg/ml), CD8^+ ^T cells were treated with a TLR4 neutralizing antibody (10 μg/ml) or chloroquine (20 μg/ml). Chloroquine prevents endosomal acidification and is a commonly-used TLR9 signaling inhibitor [31]. Chloroquine significantly reduced CSC-induced TNFα production (Figure 8A) (p < 0.0001). IL-10 production was significantly reduced by blocking either the TLR4 or TLR9 pathways (Figure 8C) (p < 0.05). As was observed in Figure 7, there was no change in IL-8 production (Figure 8B). Also, inhibition of TLR4 or TLR9 did not significantly reduce IL-1β, IL-6, IL-12p70 and IFNγ (data not shown). Collectively, our data suggest that CSC induces cytokine production from CD8^+ ^T cells through the activation of TLR4 and TLR9. This finding highlights the potential importance of TLRs on CD8^+ ^T cells in promoting inflammation in the lungs of COPD patients.

## Discussion

During the past few decades, the mortality rate of COPD has been steadily climbing and it is estimated that COPD will become the third leading cause of mortality by 2030 [32]. Therefore, current research efforts are focusing on the cellular mechanisms of COPD in an attempt to identify potential therapeutic targets. Current pharmacological treatments include bronchodilators and inhaled corticosteroids, which according to the TORCH [33] and UPLIFT [34] clinical trials, could not prevent the decline in lung function in these patients. It has been speculated that inhibiting TLRs, key cellular targets involved in initiating and maintaining an inflammatory response, could hinder the FEV_1 _decline and thus function as a therapy for COPD patients [35]. Inhibiting TLR activation would reduce the production of cytokines, thus dampening inflammation, and thereby potentially improving lung function or at least stoping its decline. Consequently, studies have investigated the expression of TLRs on both immune and epithelial cells in COPD [20,21,23,36]. There is some discrepancy between whether TLRs are up or downregulated, however this seems to vary according to the cell type being examined.

CD8^+ ^T cells are the hallmark cells of COPD and are increased in the airways of these patients. Although it is known that the cytotoxic potential of these cells is correlated to the FEV_1 _[37] and largely mediated by perforin, [38], the role of CD8^+ ^T cells in the pathogenesis of COPD still remains unclear. Our study is the first to investigate the role of TLR4 and TLR9 on CD8^+ ^T cells and we demonstrate the differential expression of TLR4 and TLR9 on CD8^+ ^T cells from COPD patients. Here, there were significantly more TLR4- and TLR9-expressing CD8^+ ^T cells isolated from the lungs of COPD patients compared to control subjects. Interestingly, there was a clear difference between lung and peripheral blood CD8^+ ^T cells in TLR expression.

Surprisingly, our initial results revealed that there was no significant difference in TLR4 and TLR9 immunoreactivity in inflammatory cells between COPD and control lung tissue. Although there were fewer inflammatory cells in our age-matched controls, almost all the immune cells expressed TLR4/TLR9. Consequently, with more cells in the COPD biopsies, fewer of these cells expressed the receptors. The fact that we observed no significant change may have been due to the variability of our samples as well as our small sample population. It is rather difficult to recruit healthy control subjects which match the age of our COPD patients and are willing to undergo bronchoscopy.

Interestingly, we found that 90% of the lung CD8^+ ^T cells expressed TLR4 and TLR9 in COPD patients, compared to only approximately 20% in control subjects. This indicates that there is a certain selectivity over which cells express these receptors in the diseased state. Under COPD conditions, the lung seems to function similarly to the intestine; the cells expressing the receptors are more specific, allowing TLR activation to become more precise, perhaps to better control persistent lung inflammation.

PBMCs from COPD patients showed increased TLR4 and TLR9 compared to control subjects. Monocytes are known to both express TLRs [14] and increase in number during chronic inflammation, and may therefore account for the elevated percentage of TLRs in PBMCs in the COPD patients. Further studies focusing on specific cell types in PBMCs, would help to clarify which cells are responsible for the increase. Contrary to the lung, the percentage of peripheral blood CD8^+ ^T cells expressing TLR4 or TLR9 was not significantly different between COPD patients and control subjects. It may be that peripheral blood CD8^+ ^T cells from COPD patients may change receptor expression once they have migrated to the lung. With approximately 2% of peripheral blood CD8^+ ^T cells expressing TLR4 or TLR9, we speculate that a trigger induces an increase (approximately 90%) in the number of CD8^+ ^T cells that express TLR4 and TLR9 in the lung. Due to the proximity of CD8^+ ^T cells to the epithelium, it is probable that cigarette smoke can cause TLR upregulation on CD8^+ ^T cells. Cigarette smoke induces inflammation in a wide variety of cell types, which may be mediated via activation of TLRs. In support of this, recent studies have shown that cigarette smoke-induced inflammation is both TLR4-dependent [39] and can induce TLR9 expression on neutrophils [40]. Here, we have demonstrated that TLR4 and TLR9 are increased by cigarette smoke exposure in CD8^+ ^T cells from COPD patients, further demonstrating that components of cigarette smoke are able to activate T cells.

It was interesting to note that CD8^+ ^T cells exposed to cigarette smoke condensate (CSC) for 24 hours did not change their TLR4 or TLR9 mRNA expression, while the protein levels of both receptors were significantly increased. These results are consistent with those from epithelial cells, which when treated for 18 hours with a 10% cigarette smoke medium did not significantly change TLR mRNA expression despite an upregulation in TLR4 protein expression [22]. When neutrophils were exposed to smoke, TLR9 mRNA expression was increased at 5 hours, but showed decreased expression at 24 hours [40]. These results, combined with our data, suggest that TLR expression may be post-transciptionally regulated in CD8^+ ^T cells.

As for the protein data, previous results have demonstrated that LPS can induce TLR4 protein expression on CD8^+ ^T cells [41], and ODN can induce TLR9 activation on CD8^+ ^T cells [42] as well as protein expression in other cells, such as neutrophils [40]. We hypothesize that the cigarette smoke is acting similarly by activating TLR4 and TLR9.

It is widely known that TLR activation results in a signal transduction cascade that acts through several pathways including NF-κB and JNK, which subsequently bind to target DNA sequences to induce cytokine expression. We hypothesized that activation of TLR4 and TLR9 by cigarette smoke would induce cytokine release by CD8^+ ^T cells. Using an established model of *in vitro *cigarette smoke exposure, CD8^+ ^T cells exposed to CSC significantly increased their levels of IL-1β, IL-6, IL-10, IL-12p70, TNFα and IFNγ, but not IL-8. Although IL-8 expression is known to be increased in COPD, our data suggest that CD8^+ ^T cells are not the major source of pulmonary IL-8 production. Lymphocytes exposed to cigarette smoke also have negligible IL-8 production compared to other peripheral blood cells [43], suggesting that the high IL-8 levels found in the lungs of COPD patients are likely derived from airway epithelial cells [44]. In addition, it has previously been reported that CD8^+ ^T cells exposed to LPS express TNFα and IFNγ in a dose-dependent manner [41], similar to our data. This implies that components in our CSC are acting as TLR ligands, activating the receptors and causing the release of cytokines. To further investigate this, we used a TLR4 neutralizing antibody and chloroquine, a TLR9 signaling inhibitor, to block both of these pathways. Chloroquine significantly reduced both TNFα and IL-10 production, whereas the TLR4 neutralizing antibody significantly inhibited IL-10. IL-10, a potent anti-inflammatory cytokine, is known to inhibit the synthesis of inflammatory cytokines, including TNFα [45]. It would not be beneficial to block a pathway that inhibits IL-10 release; however it should be noted that regulatory T cells [46] and macrophages [47] are the main producers of IL-10, not CD8^+ ^T cells. Therefore, one would have to look at the effect of these TLR inhibitors on these cells to fully understand how the use of these inhibitors would affect anti-inflammatory processes. Inhibiting TLR4 and TLR9 modestly reduced the levels of IL-1β, IL-6, IL-8, IL-12p70 and IFNγ, suggesting that other receptors play a role in the global induction of inflammatory cytokines due to smoke exposure. It is well-described that inflammatory cytokines can be produced through several pathways including, NF-κB [48] and p38 MAP kinase [49]. It is therefore not surprising that inhibiting only two of the receptors would not suppress all cytokine release. However, our findings do highlight the importance of these receptors once cigarette smoke has entered the lungs of patients.

## Conclusions

CD8^+ ^T cells are one of the most important cell types in COPD. Considering the lack of knowledge concerning the role these cells are truly playing in this disease, it is important to fully understand the phenotype of the CD8^+ ^T cells which have migrated to the lungs of these patients. We have clearly shown the distinction between the CD8^+ ^T cells expressed in the peripheral blood and those found in the lung in terms of TLR4 and TLR9 expression. Furthermore, we demonstrated for the first time that CSC is responsible for the upregulation of these receptors on lung CD8^+ ^T cells and the resulting cytokine expression. This increased cytokine expression can cause the recruitment of other inflammatory cells to the lung, further perpetuating the damage and inflammation observed in COPD patients. Blocking these receptors may prove to be useful in the future as a potential therapy.

## List of Abbreviations

COPD: chronic obstructive pulmonary disease; TLR4: toll-like receptor 4; TLR9: toll-like receptor 9; PBMC: peripheral blood mononuclear cells; CSC: cigarette smoke condensate; IL: interleukin; TNF: tumor necrosis factor; IFN: interferon; ODN: oligodeoxynucleotide

## Competing interests

The authors declare that they have no competing interests.

## Authors' contributions

JN carried out most of the experiments and drafted the manuscript. DP helped with the experiments and participated in the design of the study. JB and FM participated in the sample collection. CB and DE participated in the design of the study and helped to draft the manuscript. QH conceived the study. All authors read and approved the final manuscript.
